# Stapled peptide PROTAC induced significantly greater anti-PD-L1 effects than inhibitor in human cervical cancer cells

**DOI:** 10.3389/fimmu.2023.1193222

**Published:** 2023-05-30

**Authors:** Yu-Ying Shi, An-Jin Wang, Xue-Lian Liu, Meng-Yuan Dai, Hong-Bing Cai

**Affiliations:** ^1^ Department of Gynecological Oncology, Zhongnan Hospital of Wuhan University, Wuhan, Hubei, China; ^2^ Hubei Key Laboratory of Tumor Biological Behaviors, Wuhan, Hubei, China; ^3^ Hubei Cancer Clinical Study Center, Wuhan, Hubei, China

**Keywords:** stapled peptide PROTAC, PD-L1, inhibitor, immunotherapy, cervical cancer

## Abstract

**Introduction:**

Immune checkpoint inhibitors (ICIs) are monoclonal antibodies that target immune checkpoints that suppress immune cell activity. Low efficiency and high resistance are currently the main barriers to their clinical application. As a representative technology of targeted protein degradation, proteolysis-targeting chimeras (PROTACs) are considered to have potential for addressing these limitations.

**Methods:**

We synthesized a stapled peptide-based PROTAC (SP-PROTAC) that specifically targeted palmitoyltransferase ZDHHC3 and resulted in the decrease of PD-L1 in human cervical cancer cell lines. Flow cytometry, confocal microscopy, protein immunoblotting, Cellular Thermal Shift Assay (CETSA), and MTT assay analyses were conducted to evaluate the effects of the designed peptide and verify its safety in human cells.

**Results:**

In cervical cancer celllines C33A and HeLa, the stapled peptide strongly downregulated PD-L1 to < 50% of baseline level at 0.1 μM. DHHC3 expression decreased in both dosedependentand time-dependent manners. MG132, the proteasome inhibitor, can alleviate the SP-PROTAC mediated degradation of PD-L1 in human cancer cells. In a co-culture model of C33A and T cells, treatment with the peptide induced IFN-γ and TNF-α release in a dose-dependent manner by degrading PD-L1. These effects were more significant than that of the PD-L1 inhibitor, BMS-8.

**Conclusions:**

Cells treated with 0.1 μM of SP-PROTAC or BMS-8 for 4 h revealed that the stapled peptide decreased PD-L1 more effectively than BMS-8. DHHC3-targeting SP-PROTAC decreased PD-L1 in human cervical cancer more effectively than the inhibitor BMS-8.

## 1 Introduction

Cervical cancer is the second most widespread malignant tumor in women, with more than 500,000 new cases and 300,000 deaths annually worldwide (1–3). Patients diagnosed with early-stage cervical cancer remains a curable disease and are expected to have excellent survival outcomes, but most advanced or recurrent cervical cancer casesprognosis have rather disma prognoses (4). Effective treatments available for advanced cervical cancer are limited and are currently restricted to radiotherapy supplemented with chemotherapy (5–8). In the past several years, tumor-immunotherapy has emerged as a key candidate therapy. The cancer checkpoint blockade with inhibitors that target the programmed cell death-1/programmed cell death-ligand 1 (PD-1/PD-L1) pathway have demonstrated promising results for a wide variety of tumors (9, 10). Some PD-1/PD-L1 inhibitors have been evaluated for their effects on cervical cancer in clinical trials, revealing a degree of clinical benefits. Among these, a monotherapy using the PD-1 inhibitor pembrolizumab has been approved by the U.S. Food and Drug Administration (FDA) to treat patients with persistent, recurrent, or metastatic cervical cancer with positive PD-L1 expression, which provides hope for future clinical interventions (11, 12). However, as a single agent, pembrolizumab has limited efficacy in treating cervical cancer and should be combined with other treatments. One clinical study reported that the response rate to anti-PD-L1 treatment in patients with PD-L1 positive tumors was less than one fifth (13, 14). Furthermore, primary and acquired drug resistance gradually reduce the efficiency of immune checkpoint inhibitors (ICIs) treatment, especially in cervical cancer (15). Multiple mechanisms would result to the drug-resistance to ICIs, but PD-L1 overexpression is a common outcome in this situation (16, 17).

To improve its efficiency and overcome potential drug resistance, targeted protein degradation has been evaluated as a potential strategy (18). Proteolysis-targeting chimeras (PROTACs) have been applied in cancer treatment strategies for several years, and many of these drugs manufactured have entered the clinical experimentation stage (19). However, the limitation of this tech so far is linking small molecular drug (20). The reason for that is targeting human protein is challenging due to the difficulty of identifying kinase or protein binding pocket structure that bind small molecules. Thus, around 80% of proteins are currently considered “untargetable” or “undruggable” (21–23).Protein–protein interaction (PPI) has been easier to integrate into protein or peptide drug development, especially for intracellular targets (24). Compared to small molecules inhibitors, PROTACs bring the degradation function to the target, negating the need for an active site and expands the accessible targets well beyond those that are druggable by traditional stoichiometric inhibition (25–28). Meanwhile, peptide drugs are less toxic than small molecules inhibitors (29–31).Stapled peptides are a modified peptides by a covalent bonds to form a local alpha-helical structure. Compared to linear peptides, stapled peptides can yield improved stability, target affinity, and ability to cross the cell membrane (32–34). In our previous studies, we designed peptide-based PROTACs to decrease PD-1/PD-L1 expression (35). However, we found that targeting DHHC3, the acyltransferase of palmitoylation of PD-L1, yielded a significantly better effect than that obtained by directly targeting PD-L1 in human cancer cells. Here, we synthesized and optimized a stapled peptide to bind DHHC3 and compared its effects on decreasing PD-L1 expression in human cervical cancer cells in contrast to those of the FDA approved PD-L1 inhibitor.

## 2 Materials and methods

### 2.1 Synthesis design of peptide and characterization of stapled peptide

**Figure 1:**
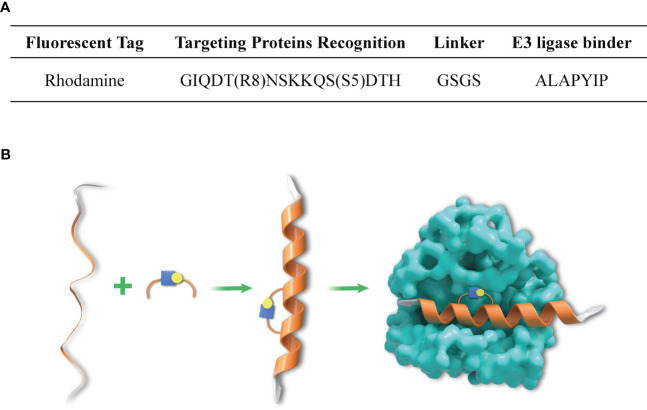
Amino acid sequence of SP-PROTAC and mechanism of DHHC3-targeting stapled peptide. **(A)** The amino acid sequence of the designed SP-PROTAC. **(B)** The linear and unconstrained peptide incorporated non-natural amino acids ((S)-N-Fmoc-2-(4´-pentenyl) alanine (S5) and (R)-N-Fmoc-2-(7´octenyl) alanine) (R8) at the respective positions, which stapled the hydrocarbon linker to improve the affinity and selectivity for targets.

The synthesis design of peptide was introduced in previous studies (25), and the characterization of stapled peptide was illustrated in **Figure 1**.

### 2.2 Cell lines

Human cervical cancer cell lines C33A and HeLa were purchased from the American Type Culture Collection (ATCC) and authenticated by short tandem repeat profiling and tested for mycoplasma contamination. Cells were maintained at 37 ˚C in 5% CO2 atmosphere. C33A was cultured in minimum essential medium (Gibco) containing 1% nonessential amino acids supplemented with 10% heat-inactivated fetal bovine serum (FBS) and 1% penicillin–streptomycin. HeLa was cultured in Dulbecco’s modified Eagle medium (Gibco) supplemented with 10% FBS and 1% penicillin–streptomycin.

### 2.3 Western blotting

C33A and HeLa cells were plated in 6-well plates and treated with the stapled peptide at the indicated concentrations for the specified time, followed by lysis in RIPA buffer containing protease inhibitor. Protein samples were prepared using a BCA Protein Assay Kit (Beyotime). A total of 50 µg of protein were added per lane, separated by 10% SDS-PAGE, and subsequently transferred onto polyvinylidene fluoride (PVDF) membrane. The membrane was blocked with 5% skimmed milk for 2 h at room temperature. Primary antibodies including anti-PD-L1 (Abcam, ab213524) and anti-GAPDH (Abcam, ab9485) antibodies were diluted in primary antibody dilution buffer (Biosharp) and incubated with PVDF membrane at 4°C overnight. Next, the membrane was incubated with goat anti-mouse/anti-rabbit secondary antibodies (Proteintech, SA00001-1, SA00001-2) at room temperature for 1 h. After washing with TBST thrice for 5 min each, the proteins were detected using enhanced chemiluminescence (ECL) reagents (Vazyme) and a ChemiDoc XRS imaging system (Bio-Rad Laboratories).

### 2.4 Flow cytometry

C33A cells were treated with the stapled peptide, digested with 0.05% trypsin (Gibco), and collected and washed with ice-cold phosphate buffered saline (PBS) two times before flow cytometry assay. The fluorescence intensity of cells was tested by a FACS Calibur (BD Biosciences) flow cytometer. For each individual experiment, 10,000 cells were counted.

### 2.5 Cellular thermal shift assay (CETSA)

C33A seeded in 6-well chamber slides were first treated with the stapled peptide and then removed and resuspended in PBS. Aliquots (500 μl) in 1.5 ml reaction tubes were exposed to various temperatures (room temperature and 37, 40, 43, 46, 49, 52, 55, 58, and 61°C) in pairs (control and peptide-treated) using a thermocycler PCR machine for 3 min, then moved to room temperature for another 3 min before being flash-frozen in liquid nitrogen and stored at −80°C. The frozen cell samples from each thermal denaturation point were lysed by adding RIPA buffer containing protease inhibitor followed by incubation on ice for 15 min and sonication at 4°C for 10 s. The lysates were centrifuged at 20,000 × g for 20 min. Western blotting was performed, and the bands were quantified as described above.

### 2.6 Immunofluorescence

The stapled peptide was labeled with rhodamine, so immunofluorescence was used to determine its distribution and interactions with the targeted POIs. After treatment with stapled peptide, the medium was removed, and cells were washed with PBS buffer. Then, 4% paraformaldehyde was added in 6-cell plates for 30 min at room temperature. Then cells were permeabilized with PBS buffer containing 0.3% Triton X-100 for 15 min at room temperature. The coverslips were incubated with 1% bovine serum albumin (BSA) for 30 min. Primary antibody anti-PD-L1 (ab213524; Abcam) diluted (1:1000) in PBS with 1% BSA was added to cells for 1 h at room temperature. Fluorescein isothiocyanate-conjugated anti-rabbit IgG was applied as the secondary antibody. Cells were counterstained with DAPI to observe the nuclei and washed three times with PBS. Anti-quenching reagent was added on glass slides, and coverslips were sealed with cells facing down on glass slides for confocal observation. Imaging was performed on a Nikon Eclipse Ni-U microscope (Nikon) equipped with Prog Res MFcool (Jenoptik AG). Digital images were processed with ImageJ (NIH).

### 2.7 T cell–C33A cell co-culture

Human peripheral blood mononuclear cells (PBMCs) were isolated from a healthy donor in an ethylenediamine tetraacetic acid anticoagulant tube by Ficoll-Paque (Sigma-Aldrich) *via* density gradient separation. CD3 T cells were isolated from PBMCs using the EasySep Human T Cell Isolation Kit (STEMCELL Technologies). C33A cells were then seeded and cocultured with the T cells at a 1:2 ratio (2.5 × 104 in 50 μl/well and 5 × 104 in 100 μl/well, respectively) in a 96-well plate at 37°C under 5% CO2. Then, SP-PROTAC and BMS-8 were added as described and maintained for 4 h. The 96-well plates were washed with PBS and stained with Hoechst 33258 (Biosharp) staining solution. The cells were washed twice and observed by fluorescence microscopy using appropriate bandpass filters.

### 2.8 Enzyme-linked Immunoassay (ELISA)

After the T cell–C33A cell co-culture assay was conducted, cell-free supernatants were collected and kept in the refrigerator at −80°C. TNF-α and IFN-γ were tested using ELISA kits (Neobioscience) following the supplier’s instructions.

### 2.9 MTT assay

Cell viability was determined by the MTT (3-(4,5-dimethylthiazol-2-yl)-2,5-diphenyltetrazoliumbromide) assay after a series of treatment. C33A and HeLa cells were collected, counted, and seeded in 96-well plates at a concentration of 5000 cells per well. After waiting 24 h for cells to adhere to the wall, cells were treated with the compounds at the indicated doses and times. Then, the medium was replaced by MTT solution at a final concentration of 0.5 mg/mL and incubated for 2 h at 37°C. Solutions were removed from the wells, and formazan crystals were dissolved in 100 μl of dimethyl sulfoxide for 10 min at 37 °C. Absorbance was read at 490 nm using a microplate reader (Perkin Elmer Inc).

### 2.10 Statistical analysis

Statistical analysis was performed using GraphPad Prism 8.1.1 software. All experiments were repeated thrice, and data were expressed as mean ± standard error of the mean (SEM). The significance of the difference between multiple groups was determined with one-way analyses of variance (ANOVA) followed by Tukey’s *post-hoc* test analysis (36). The following symbols were used: * for P<0.05, ** for P<0.01, and *** for P<0.001.

## 3 Results

**Figure 2:**
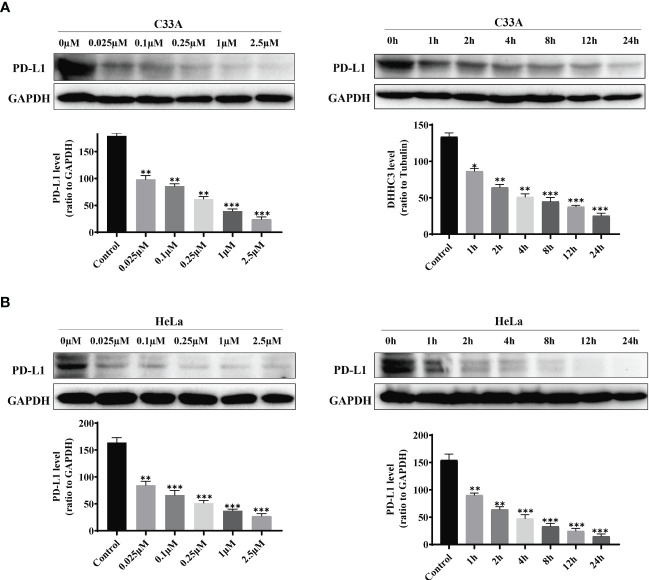
PD-L1 levels in C33A **(A)** and HeLa **(B)**cells after treatment with increasing amounts of the stapled peptide for 4 hr or 250 nM stapled peptide for the indicated times tested by western blotting assay. Gray values are presented as mean ± SEM. One-way ANOVA followed by Tukey’s *post-hoc* test compared to control (n = 3). *P < 0.05, **P < 0.01, ***P < 0.001.

**Figure 3:**
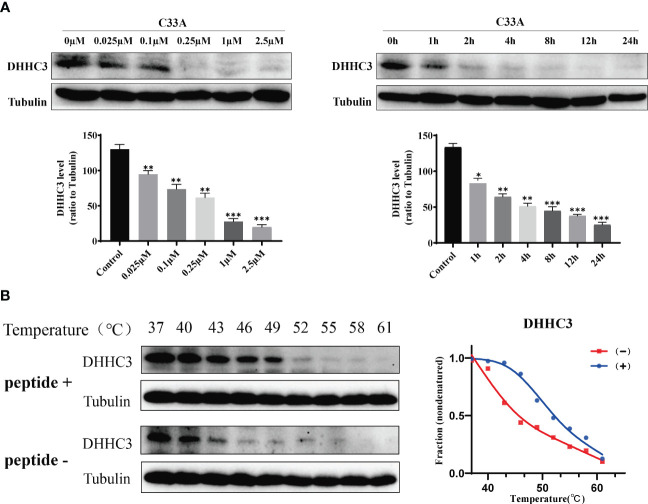
C33A cells targeted DHHC3 after treatment with the stapled peptide at indicated doses and times were immunoblotted with indicated antibodies. **(A)** Representative data set from three independent experiments. **(B)** CETSA-based determination of binding between the stapled peptide and DHHC3. CETSA curves of DHHC3 in C33A cells were determined in the absence and presence of 1 μM peptide and analyzed by western blotting. Tubulin was used as an internal control. The band intensities of DHHC3 were normalized with respect to the intensity at 40°C. **(-)** without peptide treatment, (+) with peptide treatment. *P < 0.05, **P < 0.01, ***P < 0.001.

We treated cervical cancer cell lines C33A and HeLa with the designed stapled peptide for 4 h and observed strong degradation activity against PD-L1. Moreover, we found that treatment downregulated the target protein to < 50% of baseline level at 0.1 μM. PD-L1 degradation occurred rapidly, with significant degradation occurring after 4 h of treatment with 250 nM peptide (**Figure 2**). Palmitoyltransferase ZDHHC3 (DHHC3) as the main acyltransferase required for the palmitoylation of PD-L1. When C33A was treated with the stapled peptide in the same dose and time-dependent manners, the expression of DHHC3 was gradually decreased (**Figure 3A**). To determine if the stapled peptide was able to engage with DHHC3 in the complex cellular milieu across a range of temperatures (37–61°C), lysate from C33A cells was incubated with peptide at a single compound concentration (0.1 μM). The results indicated that the peptide promoted DHHC3 stabilization at the concentrations examined (**Figure 3B**).

**Figure 4:**
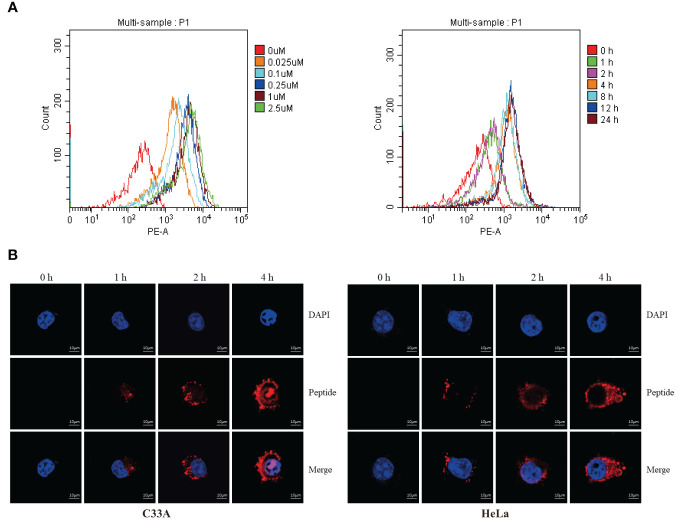
**(A)** C33A cells were incubated with rhodamine-labeled peptide targeting PD-L1 at the indicated concentrations and timepoints, then fluorescent cells were quantified by flow cytometry. Results are reported for 100,000 cells. n = 3. **(B)** Distribution of rhodamine-labeled stapled peptide in C33A and HeLa cells was observed by confocal laser microscopy in different time points. Red, Peptide; Blue, Nucleus; Scale bar, 10 μM.

**Figure 5:**
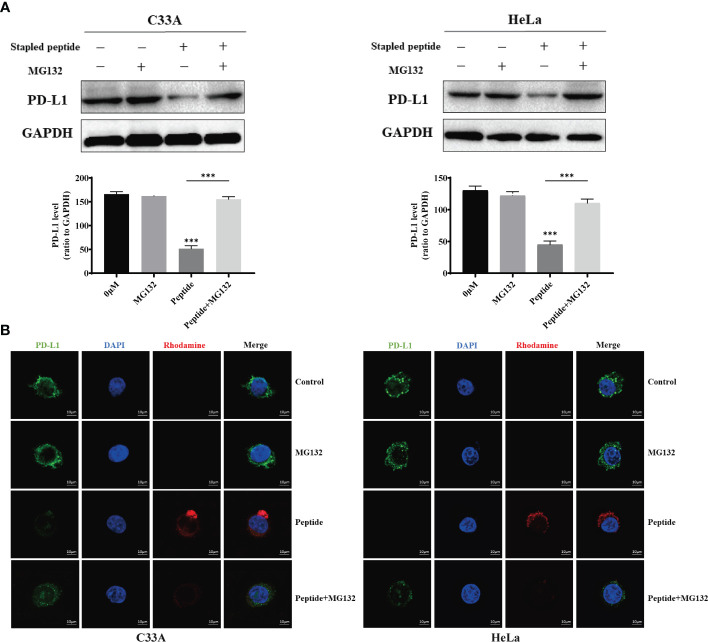
Cells were treated with 0.1 μM of rhodamine-labeled stapled peptide targeting PD-L1 in the presence or absence of MG132. **(A)** After 4 h, the PD-L1 level was detected by western blotting and quantitative analysis of the protein level in C33A and HeLa. Values are presented as mean ± SEM. One-way ANOVA followed by Tukey’s *post-hoc* test compared to control (n = 3). **(B)** After 4 h, the distribution of peptides in cells was observed by confocal laser microscopy. Red, peptide; blue, nucleus. Scale bar, 10 μM. ***P < 0.001.

Flow cytometry was used to measure fluorescence intensity, which revealed that degradation changed in a time- and dose-dependent manner (**Figure 4A**). We used electronic and confocal laser microscopy to assess whether the peptides penetrated the cell membrane, and the results demonstrated that the peptide could completely cross the cell membrane after 4 h of incubation (**Figure 4B**). In a separate experiment, we found that the proteasome inhibitor MG132 could alleviate the peptide-mediated degradation of PD-L1 in C33A and HeLa (**Figure 5A**). Moreover, the fluorescence intensity of PD-L1oberserved in cells through electronic and confocal laser microscopy was rescued with MG132 treatment, and the fluorescence intensity tendency remained corresponding with that of PD-L1 tested by western blotting (**Figure 5B**). Therefore, the data demonstrated that the stapled peptide degraded PD-L1 in a proteasome-dependent manner.

**Figure 6:**
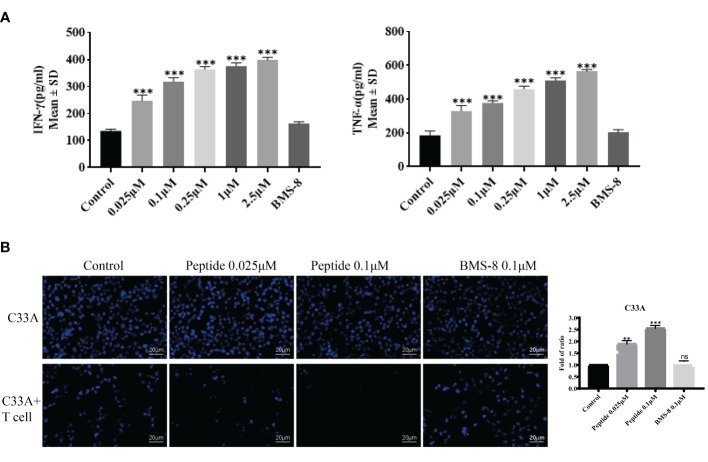
**(A)** The activity of synthesized compounds was evaluated by T cell co-culture killing assay. Effects of on IFN-γ and TNF-α secretion from T cells co-cultured with C33A cells. Values are presented as mean ± SEM. One-way ANOVA followed by Tukey’s *post-hoc* test compared to control (n = 3). **(B)** Cytotoxic effect of peptide and BMS-8 on the mortality status of C33A cells after coculture with T cells observed by flurescence microscopy on the left. Blue, nucleus. Scale bar, 10 μM. Statistical graph of T cell killing activity of peptide and BMS-8 on the right. ns, No significance, **P < 0.01, ***P < 0.001.

Because the PD-1/PD-L1 pathway is closely connected to T cell proliferation and inflammatory cytokine expression, we established a T cell–C33A cell co-culture assay to evaluate the effects of the stapled peptide. Treatment with the peptide induced IFN-γ and TNF-α release in a dose-dependent manner by degrading PD-L1, with the effects being more significant than those resulting from BMS-8 treatment (**Figure 6A**). Hoechst 33528 staining was used to measure fluorescence intensity, revealing that the stapled peptide enhanced T cell killing ability of C33A in a concentration-dependent manner after co-culture (**Figure 6B**).

**Figure 7:**
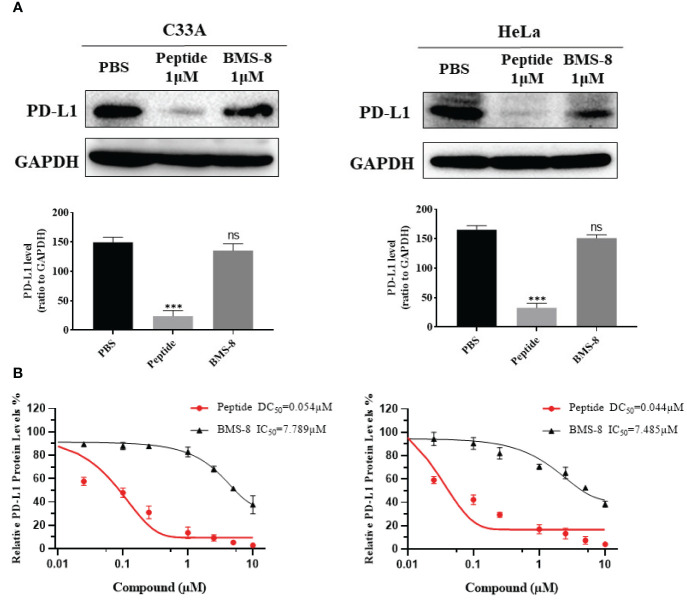
**(A)** Western blotting was conducted to reveal the effect on PD-L1 levels after treatment with peptide and BMS-8 at 0.1 μM after 4 h. Values are presented as mean ± SEM. One-way ANOVA followed by Tukey’s *post-hoc* test compared to control (n = 3). **(B)** DC50 of peptide and IC50 of BMS-8 with 4 h treatment in C33A and HeLa cells. Data are presented as mean ± SEM of 3 independent experiments. ns, No significance; ***P < 0.001.

To further confirm the effects of stapled peptide on PD-L1 degradation, we compared the PD-L1 inhibitor BMS-8 and our stapled peptide in terms of PD-L1 degradation ability in C33A and HeLa cells. Cells were treated with 0.1 μM peptide or BMS-8 induced within 4 h, the results showed the stapled peptide decreased PD-L1 more effectively than BMS-8 (**Figure 7A**). Western blotting further revealed that the stapled peptide induced PD-L1 degradation with a DC50 = 0.054 μM, while BMS-8 induced degradation with a IC50 = 7.789 μM, in C33A cells. Similarly, in HeLa cells, the stapled peptide showed a significant decrease of PD-L1 (DC50 = 0.044 μM) compared with BMS-8 the stapled peptide (IC50 = 7.485 μM). These results suggest that the stapled peptide exerted a striking downregulation effect on PD-L1 (**Figure 7B**).

**Figure 8:**
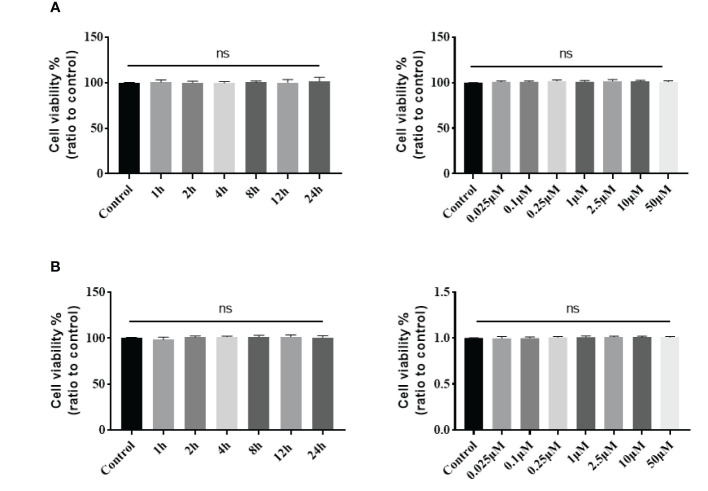
Cytotoxicity of stapled peptide in **(A)** C33A and **(B)** HeLa cells measured by the MTT assay. Data are presented as mean ± SD of three or more spheroids from three independent experiments. ns, No significance.

The stapled peptide was also tested for cytotoxicity by the MTT assay in C33A and HeLa cells. Both cell lines were exposed to increasing concentrations of the peptide for 4 h or treated with 0.1 μM for increasing periods. Our results demonstrated that the SP-PROTAC had low toxicity in C33A and HeLa cells up to a 24 h incubation time and dose of 2.5 μM (**Figure 8**).

## 4 Discussion

PROTACs are designed to utilize intracellular bioactivity mechanisms and difficult to target for the checkpoint proteins (18–20). In a previous study, we evaluated the ability of a peptide-PROTAC to degrade PD-1 and PD-L1 proteins in human cancer cells, which exhibited potent function at micro-mol/L concentrations (34). However, linear peptide–based drugs are generally unstable under *in vivo* or plasma conditions, and peptide degradation significantly limits their applicability (33). Stapled peptide modification is considered to be useful for achieving a stable and potent mini-protein structure–like binding effect with targets. The stapled peptide can be connected with an amino acid peptide linker and binder to E3 ubiquitin ligases, forming a new staple peptide–based PROTAC compound (37).

In the present study, we first synthesized a degrading compound with a stapled structure based on linear peptide binding of DHHC3 with the aim of degrading PD-L1 in human cervical cancer; the stapled peptide exhibited a potent inhibition effect. we treated human cervical cancer cells lines C33A and HeLa to asssess whether the SP-PROTAC has a more stable structure. Our results show that the modified peptide drug effectively penetrated the cell membrane. Penetration ability is one primary issue limiting the utility of peptide-based drugs as they cannot easily enter cells due to their relatively large molecular weights (26). Recently reported cell penetrating peptides (CPPs) have addressed this obstacle to some extent. The trans-activator protein Tat was first identified the function of cell membrane penetrating in HIV-1 transcription (38). Since then, numerous CPPs have been confirmed and applied in peptide-based drug development (39–41). However, there are some limitations regarding the application of CPPS: firstly, the low specificity of CPPs may result in toxicity to human cells; secondly, CPPs are relatively unstable *in vivo* before the drug arrives to the target (21). These shortcomings make them unfavorable for the clinical translation of peptide-based drugs. In contrast to CPPs, stapled peptides can better penetrate cell membranes by forming a relative stereo structure modified by the substitution or change of the net charge in the designed peptides. Moreover, the modified structure can also hide the restriction sites of linear peptide to promote stability (35). In this study, we observed a potent and stable inhibition effect as our synthesized SP-PROTAC compound effectively degraded PD-L1 at a dose of 25 nmol. Furthermore, to confirm that our observations were attributed to proteolysis degradation, we blocked the E3 ubiquitin pathway by MG132, and the decrease in PD-L1 recovered correspondingly.

Thus, we verified that our SP-PROTAC effectively limited PD-L1 in human cervical cancer cells. Because stapled peptides are a relatively new class of synthetic compounds, we tested whether the peptide itself conferred any toxicity to human cells. The MTT assay was performed to evaluate cell viability after treatment. We found that the SP-PROTAC had a low toxicity to C33A cells up to a 24 h incubation time and a dose of 50 μM. The compounds still showed significantly higher inhibition of cell-intrinsic PD-1/PD-L1 interaction in T cells and beneficial effects on T cell function. Additionally, and treatment with SP-PROTAC induced TNF-α and IFN-γ release in a dose-dependent manner, which confirmed the function of our SP-PROTAC.

Although several small molecule inhibitors have been identified to affect the PD/PD-L1 pathway, small molecules remain high challenging to utilize in drug development (21). Immobilization in the design process of SP-PROTAC improves controllability and subsequent applicability. We used an amino acid linker to connect with the binder of von Hippel-Lindau, a high-efficiency E3 ligase, which induced potent degradation for targeting proteins (18). Small molecules may be more advantageous for diversifying the selection of E3 ligases, but they are limited to relatively few binding structures in most proteins (21), which hinders their targeting ability. In our previous study, an appropriate binding peptide sequence for targeting DHHC3 was determined through PPI by a program utilizing artificial intelligence (34). Bristol-Myers Squibb (BMS) has disclosed the patent claim with structures of a number of BMS compounds, which are the potential inhibitors of the PD-1/PD-L1 pathway. One of the BMS compounds, BMS-8, binds to PD-L1 directly and induces formation of PD-L1 homodimers, which in turn prevents PD-1 interaction in previous studies (42, 43). Here, we optimized the sequence to synthesize the SP-PROTAC. The effect of our synthesized SP-PROTAC compound was much better than the conventional small molecule inhibitor of PD-L1, BMS-8 as the control to compare its effect with SP-PROTAC.

There are three main mechanisms of small molecule inhibition of PD-L1 function: blocking reaction of PD-1 to SHP2, inducing the autophagic reaction of PD-L1, and blocking PDL1/PD-1 interaction (44–47). BMS-8 can strongly bind to the monomer molecule of PD-L1 and result in homodimer formation, which inhibits the interaction between PD-1 and PD-L1 (48, 49). With a molecular weight of only 494.4, BMS-8 can work effectively on a low micro-mol/L IC50 value in some cell lines (37). Here, in both T cell function testing in co-culture model and cervical cancer lines, our synthesized SP-PROTAC compound exerted a much more potent inhibition function than that of BMS-8, especially in cancer cells, and the IC50 value of BMS-8 was more than 144–170-fold the DC50 value of the SP-PROTAC compound. Therefore, our stapled peptide may play an important role in anti-PD-1/PD-L1 pathway. Although ICIs have shown clinically significant effects in some patients with cancer, the response rate for patients with cervical cancer remains very low. In some clinical studies on patients with advanced cervical cancer, the objective response rate was not satisfactory (50, 51). To date, no small molecule inhibitors have been approved for clinical use. Considering our findings regarding safety and efficacy, the SP-PROTAC compound may have significant potential for clinical development in treating cervical cancer compared to the existing PD-L1 inhibitor.

## Data availability statement

The original contributions presented in the study are included in the article/**Supplementary Material**. Further inquiries can be directed to the corresponding authors.

## Ethics statement

The studies involving human participants were reviewed and approved by the Institutional Ethics Board of Zhongnan Hospital of Wuhan University (Wuhan, Hubei, China, No. 2020029). Written informed consent for participation was not required for this study in accordance with the national legislation and the institutional requirements.

## Author contributions

M-YD initiated and conceived the project and designed the Peptide-PROTAC drugs. H-BC wrote the manuscript and critically provided strategical advice. M-YD and Y-YS designed and performed most of the experiments and interpreted data. A-JW and X-LL provided the technical and material support. All authors contributed to the article and approved the submitted version.

## References

[1] Franco EL Schlecht NF Saslow D. The epidemiology of cervical cancer. Cancer J (2003) 9(5):34859. doi: 10.1097/00130404-200309000-00004 14690309

[2] Broutet N Jeronimo J Kumar S Almonte M Murillo R Huy NVQ. Implementation research to accelerate scale-up of national screen and treat strategies towards the elimination of cervical cancer. Prev Med (2022) 155:106906. doi: 10.1016/j.ypmed.2021.106906 34896155

[3] Arbyn M Weiderpass E Bruni L de Sanjosé S Saraiya M Ferlay J. Estimates of incidence and mortality of cervical cancer in 2018: a worldwide analysis. Lancet Glob Health (2020) 8(2):e191203. doi: 10.1016/S2214-109X(19)30482-6 PMC7025157 31812369

[4] Waggoner SE. Cervical cancer. Lancet (2003) 361(9376):221725. doi: 10.1016/S0140-6736(03)13778-6 12842378

[5] Šarenac T Mikov M. Cervical cancer, different treatments and importance of bile acids as therapeutic agents in this disease. Front Pharmacol (2019) 10:484. doi: 10.3389/fphar.2019.00484 31214018 PMC6558109

[6] Mackay HJ Wenzel L Mileshkin L. Nonsurgical management of cervical cancer: locally advanced, recurrent, and metastatic disease, survivorship, and beyond. Am Soc Clin Oncol Educ book. Am Soc Clin Oncol Annu Meeting (2015) e299–309. doi: 10.14694/EdBook_AM.2015.35.e299 PMC4920478 25993189

[7] Serkies K Jassem J. Systemic therapy for cervical carcinoma - current status. Chin J Cancer Res (2018) 30(2):20921. doi: 10.21147/j.issn.1000-9604.2018.02.04 PMC5953957 29861606

[8] Datta NR Stutz E Liu M Rogers S Klingbiel D Siebenhüner A. Concurrent chemoradiotherapy vs. radiotherapy alone in locally advanced cervix cancer: a systematic review and meta-analysis. Gynecol Oncol (2017) 145(2):37485. doi: 10.1016/j.ygyno.2017.01.033 28188016

[9] Sharma P Siddiqui BA Anandhan S Yadav SS Subudhi SK Gao J. The next decade of immune checkpoint therapy. Cancer Discov (2021) 11(4):83857. doi: 10.1158/2159-8290.CD-20-1680 33811120

[10] Guha P Heatherton KR O'Connell KP Alexander IS Katz SC. Assessing the future of solid tumor immunotherapy. Biomedicines (2022) 10(3):655. doi: 10.3390/biomedicines10030655 35327456 PMC8945484

[11] De Felice F Giudice E Bolomini G Distefano MG Scambia G Fagotti A. Pembrolizumab for advanced cervical cancer: safety and efficacy. Expert Rev Anticancer Ther (2021) 21(2):2218. doi: 10.1080/14737140.2021.1850279 33183120

[12] Qiao LY Shen S Liu M Xia C Kay JC Zhang QL. Inflammation and activity augment brain-derived neurotrophic factor peripheral release. Neuroscience (2016) 318:11421. doi: 10.1016/j.neuroscience.2016.01.018 PMC4753152 26794594

[13] Frenel JS Le Tourneau C O'Neil B Ott PA Piha-Paul SA Gomez-Roca C. Safety and efficacy of pembrolizumab in advanced, programmed death ligand 1-positive cervical cancer: results from the phase ib KEYNOTE-028 trial. J Clin Oncol (2017) 35:403541. doi: 10.1200/JCO.2017.74.5471 29095678

[14] Kennedy LB Salama AKS. A review of cancer immunotherapy toxicity. CA Cancer J Clin (2020) 70(2):86104. doi: 10.3322/caac.21596 31944278

[15] Saglam O Conejo-Garcia J. PD-1/PD-L1 immune checkpoint inhibitors in advanced cervical cancer. Integr Cancer Sci Ther (2018) 5(2):10.15761/ICST.1000272. doi: 10.15761/ICST.1000272 PMC6016855 29955379

[16] Wang Z Wu X. Study and analysis of antitumor resistance mechanism of PD1/PD-L1 immune checkpoint blocker. Cancer Med (2020) 9(21):8086121. doi: 10.1002/cam4.3410 PMC7643687 32875727

[17] Hudson K Cross N Jordan-Mahy N Leyland R. The extrinsic and intrinsic roles of PD-L1 and its receptor PD-1: implications for immunotherapy treatment. Front Immunol (2020) 11:568931. doi: 10.3389/fimmu.2020.568931 33193345 PMC7609400

[18] Dai M Radhakrishnan S Li R Tan R Yan K Fan G. Targeted protein degradation: an important tool for drug discovery for "Undruggable" tumor transcription factors. Technol Cancer Res Treat (2022) 21:15330338221095950. doi: 10.1177/15330338221095950 35466792 PMC9047787

[19] Madan J Ahuja VK Dua K Samajdar S Ramchandra M Giri S. PROTACs: current trends in protein degradation by proteolysis-targeting chimeras. Bio Drugs (2022) 36(5):60923. doi: 10.1007/s40259-022-00551-9 36098871

[20] Békés M Langley DR Crews CM. PROTAC targeted protein degraders: the past is prologue. Nat Rev Drug Discov (2022) 21(3):181200. doi: 10.1038/s41573-021-00371-6 PMC8765495 35042991

[21] Hashmi F Liu M Shen S Qiao LY. Phospholipase c gamma mediates endogenous brain-derived neurotrophic factor - regulated calcitonin gene-related peptide expression in colitis - induced visceral pain. Mol Pain (2016) 12:1744806916657088. doi: 10.1177/1744806916657088 27306412 PMC4955977

[22] Henley MJ Koehler AN. Advances in targeting 'undruggable' transcription factors with small molecules. Nat Rev Drug Discov (2021) 20(9):66988. doi: 10.1038/s41573-021-00199-0 34006959

[23] Zhuang JJ Liu Q Wu DL Tie L. Current strategies and progress for targeting the "undruggable" transcription factors. Acta Pharmacol Sin (2022) 43(10):247481. doi: 10.1038/s41401-021-00852-9 PMC9525275 35132191

[24] Au YZ Wang T Sigua LH Qi J. Peptide-based PROTAC: the predator of pathological proteins. Cell Chem Biol (2020) 27(6):6379. doi: 10.1016/j.chembiol.2020.06.002 32559499

[25] Zhou Y Liu M Li J Hashmi F Mao Z Zhang N. The impact of v-ets erythroblastosis virus E26 oncogene homolog 1 gene polymorphisms upon susceptibility to autoimmune diseases: a meta-analysis. Med (Baltimore) (2015) 94:e923. doi: 10.1097/MD.0000000000000923 PMC4616355 26039128

[26] Zhao L Zhao J Zhong K Tong A Jia D. Targeted protein degradation: mechanisms,strategies and application. Signal Transduct Target Ther (2022) 7(1):113. doi: 10.1038/s41392-022-00966-4 35379777 PMC8977435

[27] Yang J Gao C Liu M Liu YC Kwon J Qi J. Targeting an inducible SALL4-mediated cancer vulnerability with sequential therapy. Cancer Res (2021) 81(23):601828. doi: 10.1158/0008-5472.CAN-21-0030 PMC8639708 34593523

[28] Schneider M Radoux CJ Hercules A Ochoa D Dunham I Zalmas LP. The PROTAC table genome. Nat Rev Drug Discov (2021) 20(10):78997. doi: 10.1038/s41573-021-00245-x 34285415

[29] Yan S Yan J Liu D Li X Kang Q You W. A nano-predator of pathological MDMX construct by clearable supramolecular gold(I)-thiol-peptide complexes achieves safe and potent anti-tumor activity. Theranostics (2021) 11(14):683346. doi: 10.7150/thno.59020 PMC8171083 34093856

[30] McGill AR Kahlil R Dutta R Green R Howell M Mohapatra S. SARS-CoV-2 immuno-pathogenesis and potential for diverse vaccines and therapies: opportunities and challenges. Infect Dis Rep (2021) 13(1):10225. doi: 10.3390/idr13010013 PMC7931091 33557330

[31] Ke Y Weng M Chhetri G Usman M Li Y Yu Q. Trappc9 deficiency in mice impairs learning and memory by causing imbalance of dopamine D1 and D2 neurons. Sci Adv (2020) 6(47):eabb7781. doi: 10.1126/sciadv.abb7781 33208359 PMC7673810

[32] Atangcho L Navaratna T Thurber GM. Hitting undruggable targets: viewing stabilized peptide development through the lens of quantitative systems pharmacology. Trends Biochem Sci (2019) 44(3):24157. doi: 10.1016/j.tibs.2018.11.008 PMC6661118 30563724

[33] Böttger R Hoffmann R Knappe D. Differential stability of therapeutic peptides with different proteolytic cleavage sites in blood, plasma and serum. PloS One (2017) 12(6):e0178943. doi: 10.1371/journal.pone.0178943 28575099 PMC5456363

[34] Dai MY Shi YY Wang AJ Liu XL Liu M Cai HB. High-potency PD-1/PD-L1 degradation induced by peptide-PROTAC in human cancer cells. Cell Death Dis (2022) 13(11):924. doi: 10.1038/s41419-022-05375-7 36333311 PMC9636179

[35] Guenette RG Yang SW Min J Pei B Potts PR. Target and tissue selectivity of PROTAC degraders. Chem Soc Rev (2022) 51(14):574056. doi: 10.1039/D2CS00200K 35587208

[36] Liu C Cripe TP Kim MO. Statistical issues in longitudinal data analysis for treatment efficacy studies in the biomedical sciences. Mol Ther (2010) 18(9):172430. doi: 10.1038/mt.2010.127 PMC2956920 20588256

[37] Liao H Li X Zhao L Wang Y Wang X Wu Y. A PROTAC peptide induces durable b-catenin degradation and suppresses wnt-dependent intestinal cancer. Cell Discov (2020) 6:35. doi: 10.1038/s41421-020-0171-1 32550000 PMC7280531

[38] Green M Loewenstein PM. Autonomous functional domains of chemically synthesized human immunodeficiency virus tat trans-activator protein. Cell (1988) 55:117988. doi: 10.1016/0092-8674(88)90262-0 2849509

[39] Berillo D Yeskendir A Zharkinbekov Z Raziyeva K Saparov A. Peptide-based drug delivery systems. Medicina (Kaunas) (2021) 57(11):1209. doi: 10.3390/medicina57111209 34833427 PMC8617776

[40] Ke Y Bu S Ma H Gao L Cai Y Zhang Y. Preventive and therapeutic effects of astaxanthin on depressive-like behaviors in high-fat diet and streptozotocin-treated rats. Front Pharmacol (2020) 10:1621. doi: 10.3389/fphar.2019.01621 32082151 PMC7003134

[41] Varanko A Saha S Chilkoti A. Recent trends in protein and peptide-based biomaterials for advanced drug delivery. Adv Drug Deliv Rev (2020) 156:13387. doi: 10.1016/j.addr.2020.08.008 PMC7456198 32871201

[42] Kim EH Kawamoto M Dharmatti R Kobatake E Ito Y Miyatake H. Preparation of biphenyl-conjugated bromotyrosine for inhibition of PD-1/PD-L1 immune checkpoint interactions. Int J Mol Sci (2020) 21(10):3639. doi: 10.3390/ijms21103639 32455628 PMC7279355

[43] Musielak B Kocik J Skalniak L Magiera-Mularz K Sala D Czub M. CA-170 - a potent small-molecule PD-L1 inhibitor or not? Molecules (2019) 24(15):2804. doi: 10.3390/molecules24152804 31374878 PMC6695792

[44] Zhou M Zou X Cheng K Zhong S Su Y Wu T. The role of cell-penetrating peptides in potential anti-cancer therapy. Clin Transl Med (2022) 12(5):e822. doi: 10.1002/ctm2.822 35593206 PMC9121317

[45] Li R Liu M Yang Z Li J Gao Y Tan R. Proteolysis-targeting chimeras (PROTACs) in cancer therapy: present and future. Molecules (2022) 27(24):8828. doi: 10.3390/molecules27248828 36557960 PMC9785308

[46] Chen L Ke Y Ma H Gao L Zhou Y Zhu H. Fluoxetine and ketamine reverse the depressive but not anxiety behavior induced by lesion of cholinergic neurons in the horizontal limb of the diagonal band of broca in male rat. Front Behav Neurosci (2021) 15:602708. doi: 10.3389/fnbeh.2021.602708 33679340 PMC7930217

[47] Wu Q Jiang L Li SC He QJ Yang B Cao J. Small molecule inhibitors targeting the PD-1/PD-L1 signaling pathway. Acta Pharmacol Sin (2021) 42(1):19. doi: 10.1038/s41401-020-0366-x PMC7921448 32152439

[48] Shi D An X Bai Q Bing Z Zhou S Liu H. Computational insight into the small molecule intervening PD-L1 dimerization and the potential structure-activity relationship. Front Chem (2019) 7:764. doi: 10.3389/fchem.2019.00764 31781546 PMC6861162

[49] Skalniak L Zak KM Guzik K Magiera K Musielak B Pachota M. Small-molecule inhibitors of PD-1/PD-L1 immune checkpoint alleviate the PD-L1-induced exhaustion of t-cells. Oncotarget (2017) 8(42):7216781. doi: 10.18632/oncotarget.20050 PMC5641120 29069777

[50] Ge Y Zhang Y Zhao KN Zhu H. Emerging therapeutic strategies of different immunotherapy approaches combined with PD-1/PD-L1 blockade in cervical cancer. Drug Des Devel Ther (2022) 16:305570. doi: 10.2147/DDDT.S374672 PMC9470119 36110399

[51] Solorzano-Ibarra F Alejandre-Gonzalez AG Ortiz-Lazareno PC Bastidas-Ramirez BE Zepeda-Moreno A Tellez-Bañuelos MC. Immune checkpoint expression on peripheral cytotoxic lymphocytes in cervical cancer patients: moving beyond the PD-1/PD-L1 axis. Clin Exp Immunol (2021) 204(1):7895. doi: 10.1111/cei.13561 PMC7944364 33306195

