# Supplementary material for: Stapled peptide PROTAC induced significantly greater anti-PD-L1 effects than inhibitor in human cervical cancer cells
Source: Front Immunol. 2023 May 30;14:1193222. doi: 10.3389/fimmu.2023.1193222 (PMC10262918; doi:10.3389/fimmu.2023.1193222)
Supplement: Supplementary file 1 [file DataSheet_1.docx]

Raw Data for

**Stapled peptide PROTAC induced significantly greater anti-PD-L1 effects than inhibitor in human cervical cancer cells**

The original data is too large to upload from the upload path of the Additional files, so we generate the following link from the original data.

**Links to raw data:**

[**https://www.jianguoyun.com/p/Dcvy1BYQ_fe_Cxi33P4EIAA**](https://www.jianguoyun.com/p/Dcvy1BYQ_fe_Cxi33P4EIAA)

The raw data includes four folders:

**1. Supplementary data sheet:**


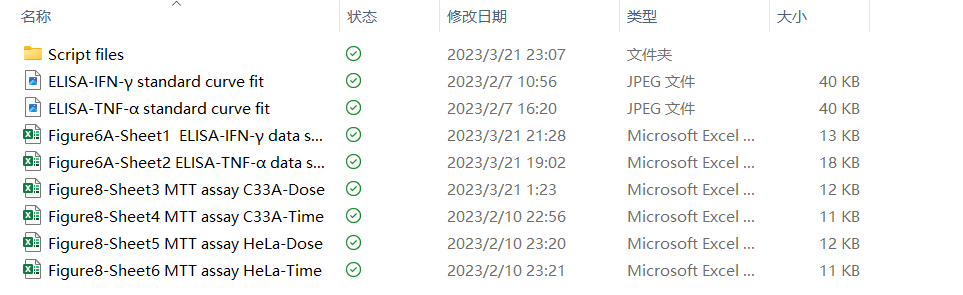
This section includes script files and data sheets for Figure 6A and Figure 8.


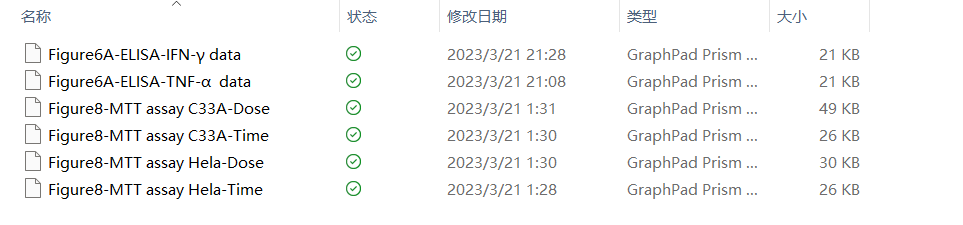
The folder named as “script files” includes statistical script files used for the data analysis of ELISA and MTT assays.

**2. Supplementary flow cytometry data**

This section includes flow cytometry data (.fcs) included in Figure 4A.


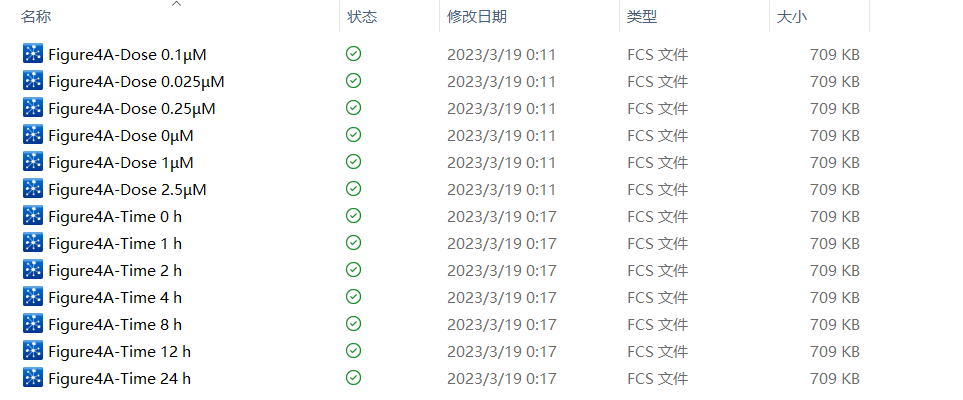
**3. Supplementary image**


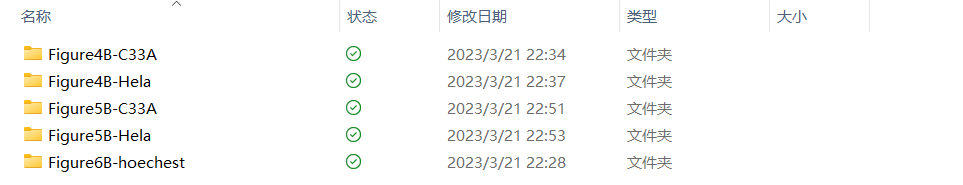
This section includes five folders with images in Figure 4B、5B and 6B.

**4. Supplementary WB raw data**


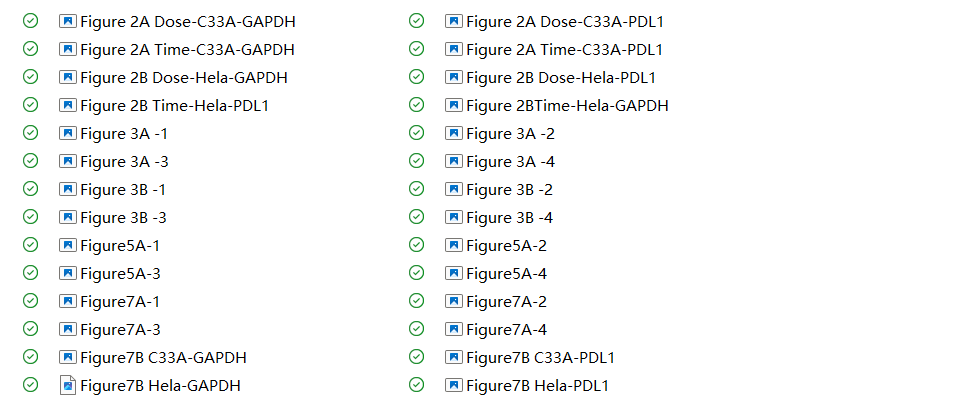
This section includes Western Blot raw data in Figure 2、Figure3、Figure5 and Figure7.
